# Contribution of Saudi Neurosurgeons to the International Neurosurgical Literature: a Bibliometric Analysis of Trends Over the Last Three Decades

**DOI:** 10.7759/cureus.35164

**Published:** 2023-02-19

**Authors:** Abdulhakim B Jamjoom, Abdulhadi Y Gahtani, Omar M Jamjoom, Yousef K Khogeer, Momen Sharab, Moajeb T Alzahrani

**Affiliations:** 1 Neurological Surgery, King Saud bin Abdulaziz University for Health Sciences, Jeddah, SAU; 2 Neurological Surgery, King Khalid National Guards Hospital, Jeddah, SAU; 3 Medicine, University of Central Lancashire, Preston, GBR

**Keywords:** citation rates, pubmed, publication trends, bibliometrics, neurosurgical journals, neurosurgery, saudi arabia

## Abstract

This review is a bibliometric analysis of the contribution of neurosurgeons from the Kingdom of Saudi Arabia (KSA) to the international neurosurgical literature over the last three decades. The study aimed at determining changes in publication trends over time and assessing the impact of these changes on citation numbers. All publications in the PubMed-indexed neurosurgical journals that were authored by at least one Saudi neurosurgeon were selected. The articles were divided into two study groups according to publication year whether during the last decade (2011- 2020) or the previous two decades (1991- 2010). Changes in publication trends were determined by comparing the bibliometric characteristics of the articles in both groups. The impact of the changes on citation numbers was assessed by correlating the annual citation rates for the articles with their bibliometric qualities.

A total of 352 publications were suitable for the review (200 articles published during 2011- 2020, and 152 during 1991- 2010). Temporal changes in the publishing journals and first authors’ centres and regions were observed. The articles that were published in the last decade were associated with a significantly higher annual publication rate, a greater number of authors, centres, and countries, and a larger sample size compared to those published in the previous two decades. They also had a lower percentage of Saudi total and first authorship as well as a smaller proportion of case reports. The annual citation rate was significantly impacted by the duration from publication, sample size, and study type during both study periods. However, only during the last decade, the annual citation rate was positively influenced by the journal’s impact factor, number of authors, centres, countries, and percentage of Saudi authorship.

We conclude that KSA neurosurgeons’ contribution to international neurosurgical journals had increased considerably over the last decade. The publications were authored by neurosurgeons from a wider range of centres and regions than in the past. A bigger portion of publications had become more multi-authored, multi-centred, and multi-national as well as reported larger sample sizes and lesser rates of case reports. The changes in publication trends correlated positively with the articles’ annual citation rates. The findings could be considered encouraging.

## Introduction and background

Academic productivity is the hallmark of a successful career in any medical field. Bibliometrics is the use of statistical methods to examine the literature quantitatively in terms of output and impact on future research [1]. The popularity of bibliometric studies had grown in sync with the surge in scientific productivity in recent years [2]. Bibliometric tools are increasingly being utilized in practices such as evaluating grant allocation, employment, promotion, awards, and fellowships in scientific societies [3]. Citation analysis, which is the assessment of the frequency and patterns of articles, is one of the most commonly used measures. Other parameters include the total number of publications, number of authors, number of institutions, number of countries, publishing journal’s impact factor (IF), study population size, study type, and level of evidence (LOE) [4-6]. 

Over the years, researchers in the Kingdom of Saudi Arabia (KSA) contributed substantially to the national and international medical literature [1]. The academic productivity of the country grew by 14.1% per year from 2008 to 2017 [7]. The contribution of researchers from KSA to the neurosciences literature was the focus of two bibliometric analyses in recent years [8,9]. Alhibshi et al. [8] reported a rise in the number of publications from 123 in 2013 to 332 in 2018. Bardeesi et al. [9] calculated the country’s clinical neurosciences research global ranking during 1996-2018 as 38th for total documents, 40th for total citations, and 132nd for citations per document. The relatively low citations per document ranking was attributed to researchers publishing in local and low IF journals [9].

In the field of neurosurgery, bibliometric review of the contribution of different countries to the speciality’s international journals is topical [3]. The input of neurosurgeons from KSA to the literature had been the subject of a few studies [10-12]. These included an analysis of the most cited KSA neurosurgical publications [10], a survey of the h-index for Saudi neurosurgeons [11], and a grading of the LOE of clinical neurosurgery research in KSA [12]. The latter focused on articles that were published in medical and neurosciences journals.

At present, the literature lacks up-to-date data that assess the contribution of neurosurgeons from KSA to international neurosurgical journals. This review is a bibliometric evaluation of these publications over the last three decades. It aimed at determining the changes in publication trends over time and assessing the impact of these changes on citation patterns.

## Review

Methods

Institutional Review Board (IRB) approval was obtained from King Abdulla International Medical Research Centre, Ministry of National Guards, KSA (approval number: IRB/2330/22). The PubMed database was searched on August 1, 2022, for suitable articles using the following combinations: [Affiliation] Saudi Arabia OR Kingdom of Saudi Arabia AND [Affiliation] Neurosurgery OR Neurological Surgery AND [Journal] individual international neurosurgical journals by name.

The following journals were searched: Journal of Neurosurgery, Journal of Neurosurgery: Spine, Journal of Neurosurgery: Pediatrics, World Neurosurgery, Neurosurgery, Acta Neurochirurgica, British Journal of Neurosurgery, Surgical Neurology, Surgical Neurology International, Journal of Neurology Neurosurgery and Psychiatry, Neurosurgical Focus, Neurosurgical Review, Pediatric Neurosurgery, Journal of Neurosurgical Sciences, Clinical Neurology and Neurosurgery, Clinical Neurosurgery, Neurosurgery Clinics of North America, Journal of Neurological Surgery Part A: Central European Neurosurgery, Journal of Neurological Surgery Part B: Skull Base, Stereotactic and Functional Neurosurgery, Neurochirugie, Operative Neurosurgery, Asian Journal of Neurosurgery, Child’s Nervous System, Journal of Craniovertebral Junction and Spine, Pituitary, Spine Journal, Joint Bone Spine, Spine (Phila Pa 1976), Neurospine, European Spine Journal, Spinal Cord, and Journal of Neurotrauma.

The inclusion criteria were articles that were published in international neurosurgical journals that had at least one author affiliated to a neurosurgical centre in KSA. All types of publications were included except communications and letters to editors. Using abstracts or full articles, when necessary, the following data were collected: year of publication, publishing journal, its IF, number of authors, number of KSA authors, number of KSA first authors, their centres, their regions, percentage of KSA authors, number of centres, and number of countries. The journal's IF data was obtained from the International Scientific Institute website [13]. A Saudi neurosurgeon was defined as any author affiliated to a centre in KSA irrespective of his/her nationality. The percentage of KSA authors was calculated by dividing the number of KSA authors by the total number of authors and expressed as a percentage. Other data that was gathered included study sample size (if available), and study type (whether case report, case series, prospective study, review, experimental study, survey, cross-sectional study, or guidelines). Using Google Scholar (Google LLC, Mountain View, California, United States), the citation numbers for each article were obtained. In view of the regular changes in citation records, the search findings on a single day (September 1, 2022) were documented and used for analysis. For each article, the number of citations was divided by the duration from publication in years to calculate the annual citation rate.

The changes in the publication trends over time were assessed by comparing the bibliometric parameters for articles that were published over the last decade (2011-2020) with articles that were published during the previous two decades (1991-2010). Data for publications during 1991-2010 were analysed together as the focus of the study was the country's contribution to the international neurosurgical literature in the last decade and separating the dates of the previous two decades into two (1991-2000 and 2001-2010) would not have served the objectives of the study. The following factors were used: annual publication rate, journal’s IF, number of authors, number of first authors from KSA, percentage of authors from KSA, number of centres, number of countries, sample size, study type, number of citations, and the annual citation rate. The findings for the two study periods were examined statistically using the mean difference (MD) test from an online source [14] or the chi-squared (X2) test from an online source [15]. The impact of the variation in publication trends on citation numbers was assessed by correlating the annual citation rates with various bibliometric parameters’ data for both study periods using the Spearman’s Rho correlation coefficient test from an online source [15].

Results

**Figure 1:**
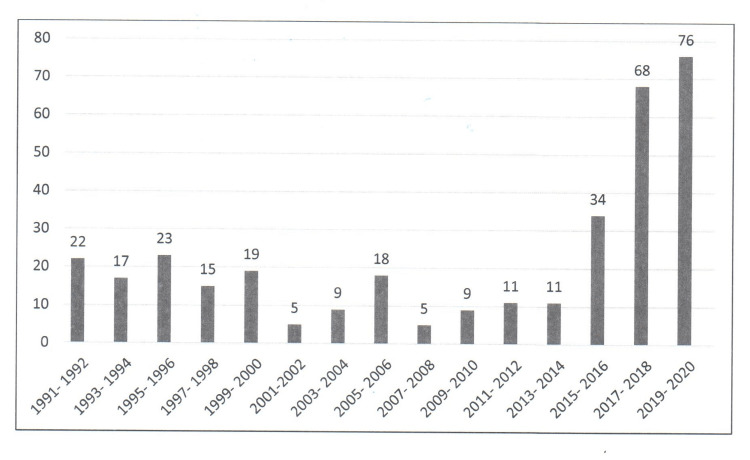
The two-yearly number of KSA publications in the international neurosurgical literature over the last three decades (1991-2020) KSA: Kingdom of Saudi Arabia

A total of 352 articles published by Saudi neurosurgeons in international neurosurgical journals during the last three decades were suitable and included in this review. The number of articles that were published in the last decade (2011- 2020) was 200. The number of articles that were published during the previous two decades (1991- 2010) was 152. The median (range) number of publications per year was eight (1-47). The annual publication rate fluctuated during 1991-2014, but the number of articles had been steadily growing since 2015 (Figure 1).

**Figure 2:**
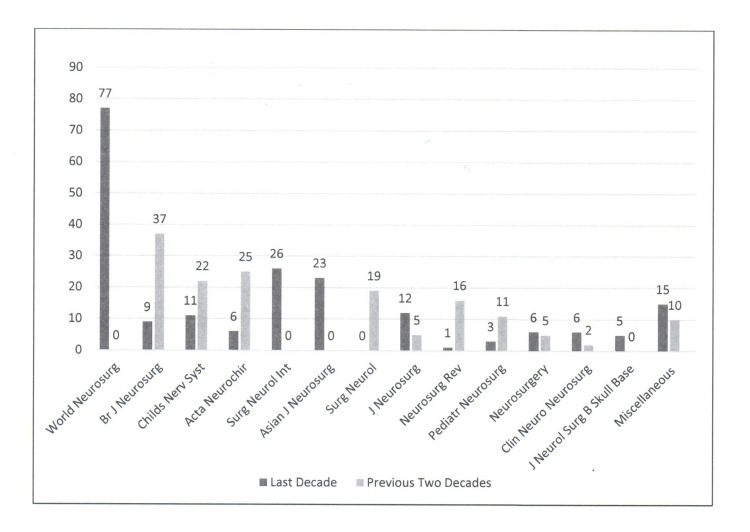
The distribution of KSA publications in the international neurosurgical literature during the last decade (2011- 2020) and the previous two decades (1991-2010) according to their publishing journal KSA: Kingdom of Saudi Arabia

The distribution of articles according to their publishing journals is demonstrated in Figure 2.

A temporal change in the most common publishing journals was noted. During 1991-2010, the journals with the highest number of publications were the British Journal of Neurosurgery: 37 (24%), Acta Neurochirurgica: 25 (16%), Childs Nervous System: 22 (15%), and Surgical Neurology: 19 (13%). However, during 2011-2020, most articles were published in World Neurosurgery: 77 (39%), Surgical Neurology International: 26 (13%), Asian Journal of Neurosurgery: 23 (12%), and Journal of Neurosurgery: 12 (6%).

The findings relating to several bibliometric parameters are summarized in Table 1.

**Table 1:** The median (range), mean (±SD) and mean difference comparative analysis for several bibliometric parameters relating to the KSA publications in the international neurosurgical journals during 2011-2020 (200 articles) and 1991-2010 (152 articles) *Denotes significance KSA: Kingdom of Saudi Arabia; IF: impact factor

Parameters		2011-2020	1991-2010	Mean Difference (95% CI)	P-value
Annual publication rate	Median (range)	17 (3- 47)	7.5 (1- 19)		
	Mean (±SD)	20 (±15.7)	7.6 (±4.6)	14.4 (4.7- 20.1)	0.0026*
Journal’s IF	Median (range)	2.1 (0- 10.15)	1.6 (0.86- 5.12)		
	Mean (±SD)	1.96 (±1.4)	2.04 (±1.06)	0.08 (0.19- 0.35)	0.5570
Number of authors	Median (range)	5 (1- 62)	3 (1- 8)		
	Mean (±SD)	6.4 (±6)	3.3 (±1.7)	3.1 (2.1- 4.1)	<0.0001*
Percentage of KSA authors	Median (range)	66% (3%- 100%)	100% (14%-100%)		
	Mean (±SD)	61% (±39%)	93% (±21%)	32% (25%- 39%)	<0.0001*
Number of centres	Median (range)	3 (1- 34)	1 (1- 4)		
	Mean (±SD)	3.4 (±3.6)	1.1 (±0.4)	2.2 (1.7- 2.8)	<0.0001*
Number of countries	Median (range)	2 (1-15)	1 (1- 2)		
	Mean (±SD)	1.9 (±31.3)	1.1 (±30.3)	0.8 (0.6- 1)	<0.0001*
Sample size	Median (range)	4.5 (1- 1848)	1 (1- 633)		
	Mean (±SD)	74 (±238.2)	22.5 (±75.6)	51.4 (10.4- 92.5)	0.0142*
Numbers of citations	Median (range)	7 (0- 180)	19.5 (0- 240)		
	Mean (±SD)	14 (± 21)	29 (± 30)	15 (9.7- 20.3)	<0.0001*
Duration from publication (years)	Median (range)	4 (2-11)	25 (12- 31)		
	Mean (±SD)	4.5 (± 2.7)	23.6 (± 5.5)	19.1 (11- 18.2)	<0.0001*
Number of citations per article per year	Median (range)	1.7 (0- 31.5)	0.8 (0- 17.1)		
	Mean (±SD)	3 (± 3.8)	1.4 (± 1.8)	1.6 (0.9- 2.32)	<0.0001*

Over the last decade, compared to the previous two decades, articles from KSA that were published in international neurosurgical journals had a significantly higher means of annual publication rate (20 versus (vs.) 7.6) (P=0.0026), number of authors (6.4 vs. 3.3) (P<0.0001), number of centres (3.4 vs. 1.1) (P<0.0001), number of countries (1.9 vs. 1.1) (P<0.0001), sample size (74 (164 articles) vs. 22.5 (142 articles)) (P=0.0142) and annual citation rate (3 vs. 1.4) (P<0.0001). They also had a significantly lower percentage of KSA authors (61% vs. 93%) (P<0.0001) and lower mean citation numbers (14 vs. 29) (P<0.0001). A slight reduction in the mean journal’s IF was observed as well but this did not reach significance (1.96 vs. 2.04) (P=0.5570).

Table 2 shows the findings involving two parameters. The KSA first author rate was significantly lower in publications during 2011-2020 compared to 1991-2010 (71% vs. 100%) (P<0.0001). At the same time, there was a significant change in study type with a decrease in the rates of case reports (41% vs. 59.2%) and an increase in review articles (15.5% vs. 4.6%) (P=0.002).

**Table 2:** The findings and X2 comparative analysis for KSA publications in the international neurosurgical literature during 2011-2020 (200 articles) and 1991-2010 (152 articles) *Denotes significance, **Technical, guidelines, surveys, cross-sectional X2: Chi-squared; KSA: Kingdom of Saudi Arabia

Parameters		2011-2020	1991-2010	X2 -value	P -value
		Articles number	Articles number		
KSA author role	First author	141 (71%)	152 (100%)	50.81	<0.0001*
	Co-author	59 (28%)	0		
Study Type	Case reports	82 (41%)	90 (59.2%)	16.94	0.002*
	Case series	70 (35%)	43 (28.3%)		
	Reviews	31 (15.5%)	7 (4.6%)		
	Prospective	4 (2%)	4 (2.6%)		
	Experimental	8 (4%)	4 (2.6%)		
	Others**	5 (2.5%)	4 (2.6%)		

**Figure 3:**
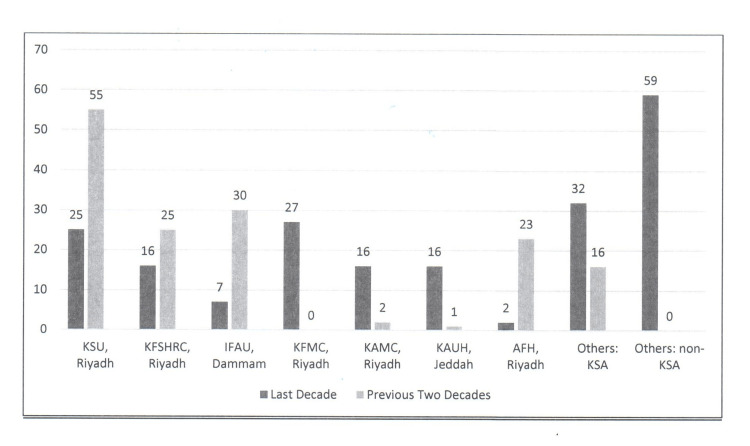
The distribution of KSA publications in the international neurosurgical literature during the last decade (2011-2020) and the previous two decades (1991-2010) according to the first author’s KSA centre KSA: Kingdom of Saudi Arabia; KSU: King Saud University, Riyadh; KFSHRC: King Faisal Specialist Hospital and Research Centre, Riyadh; IFAU: Imam Faisal bin Abdulrahman University Hospital, Dammam; KFMC: King Fahad Medical City, Riyadh; KAMC: King Abdulaziz Medical City, Riyadh; KAUH: King Abdulaziz University Hospital, Jeddah; AFH: Armed Forces Hospital, Riyadh

The distribution of publications from KSA according to the first author’s centre is illustrated in Figure 3. Some time-based variation was observed. During 1991-2010, the majority of KSA first authors were from King Saud University, Riyadh (KSU): 55 (36.5%), Imam Faisal bin Abdulrahman University Hospital, Dammam (IFAU): 30 (19.7%), King Faisal Specialist Hospital and Research Centre, Riyadh (KFSHRC): 25 (16.5%), and Armed Forces Hospital, Riyadh (AFH): 23 (15.1%). However, during 2011-2020, the KSA first authors were largely from King Fahad Medical City, Riyadh (KFMC): 27 (13.5%), KSU: 25 (12.5%), KFSHRC: 16 (8%), King Abdulaziz University Hospital, Jeddah (KAU): 16 (8%), and King Abdulaziz Medical City, Riyadh (KAMC): 16 (8%).

**Figure 4:**
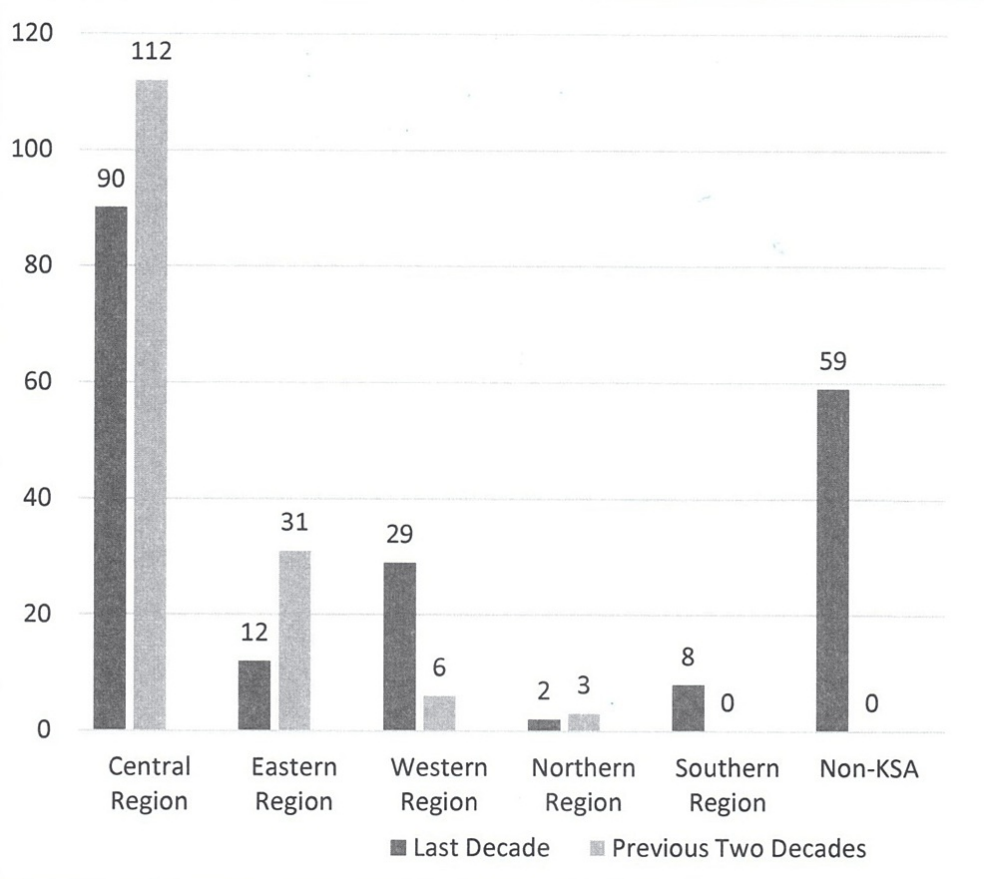
The distribution of KSA publications in the international neurosurgical literature during the last decade (2011-2020) and the previous two decades (1991-2010) according to the lead author’s institution KSA region KSA: Kingdom of Saudi Arabia

The distribution of publications according to the first author’s KSA region is shown in Figure 4. A slight deviation was seen over the years. During the previous two decades (1991-2010), the first authors of most of the publications were from the central region: 112 (73.7%) and the eastern region: 31 (20.4%). However, over the last decade (2011-2020), the proportion of first authorship became central region: 90 (45%), western region: 29 (14.5%), and eastern region: 12 (6%).

Table 3 illustrates the correlation analysis between the annual citation rate and data relating to several bibliometric parameters. A positive correlation was observed between the annual citation rate and duration from publication, sample size, and study type in both study periods (2011-2020 and 1991-2010). However, only in publications over the last decade (2011-2020), the annual citation rate was significantly impacted by the journal’s IF (P=0), number of authors (P<0.0001), number of centres (P=0.0166), number of countries (P=0.0041), and percentage of authors from KSA (P=0.0004). The rate of first authors from KSA had no impact on annual citation rates in both study periods.

**Table 3:** Spearman’s Rho (rs) correlation analysis between the articles’ annual citations rates and several parameters for Saudi publications in the international neurosurgical literature in 2011- 2020 (200 articles) and 1991- 2010 (152 articles) KSA: Kingdom of Saudi Arabia

Parameters	Last Decade (2011-2020)		Previous Two Decades (1991-2010)	
	r_s_ -value	P-value	r_s_-value	P-value
Duration from publication in years	0.1515	0.03*	0.2593	0.0013*
Publishing journal’s impact factor	0.3389	0*	0.1146	0.1599
Number of authors	0.2787	<0.0001*	0.0342	0.6758
Number of centres	0.1693	0.0166*	0.0369	0.6516
Number of countries	0.2019	0.0041*	0.0571	0.4845
Percentage authorship from KSA	-0.2486	0.0004*	-0.0756	0.3547
First authorship from KSA	0.1362	0.0545	0.0316	0.6995
Sample size	0.4721	0*	0.2331	0.0053*
Study type	0.5048	0*	0.2525	0.0017*
*Denotes significance at P ≤0.05				

Discussion

This study was a review of 352 Saudi publications in PubMed-indexed neurosurgical journals during 1991-2020. The country’s neurosurgeons had published 377 articles in the medical and neurosciences journals during 1990-2013 [12]. KSA’s contribution to the neurosurgical literature (352 articles in 30 years) was higher than what was published during 1989-2018 by countries such as Morocco (174), Portugal (146), Argentina (210), and Egypt (338) [3]. The mean annual publication rate had increased significantly from 7.6 in 1991-2010 to 20 in 2011-2020. This proliferation was comparable to the observed global 2.5-fold growth in neurosurgical productivity in six major neurosurgical journals [16]. The observed rise in neurosurgical productivity was probably influenced by the increase in the number of neurosurgeons in the country of late [16,17]. The mean neurosurgeon density in KSA was calculated in 2021 as 7.5 per million population [17]. The output was possibly impacted by the yield of individual neurosurgeons and neurosurgical centres, which are known to vary considerably. The h-index for 84 KSA neurosurgeons was reported as ranging from 0 to 33 in 2015 [11], while the h-index for 20 UK neurosurgical units ranged from 4 to 13 in 2016 [18]. Furthermore, the upsurge in productivity was possibly affected by a few of the country-specific characteristics which had changed over time [19]. In addition to the increase in the KSA population [20], the number of KSA universities in the world's top 500 increased from zero in 2004, to two in 2011, and to five in 2022 [21]. The number of KSA clinical neurology journals in SJR changed from zero in 2001 to one in 2022 [22], and the percentage of gross domestic product (GDP) spent on research and development (R&D) by KSA grew from 0.06% in 2003 to 0.52% in 2020 [23].

A change in the most common publishing journals was noted. The shift was probably influenced by the emergence of new journals, the training background of authors, as well as the scientific strength of the articles. Over the last decade (2011-2020), most of the articles were published in World Neurosurgery and Surgical Neurology International, which were launched in 2010 and 2011, respectively [13]. During the previous two decades (1991-2010), the most frequently used journals were the British Journal of Neurosurgery and Acta Neurochirurgica, which were launched in 1987 and 1950, respectively [13]. The change in the utilized journals, however, did not indicate a significant change in trends in the journal’s IF over the years.

Publications from KSA in international neurosurgical journals had a significantly higher mean number of authors, centres, and countries during 2011-2020, compared to 1991-2010. This signifies that the country’s neurosurgical research had become more collaborative, multi-authored, multi-centred, and multi-national. This is not unusual as the neurosurgical community in KSA is relatively young [17], and research cooperation had increased in recent years, largely amongst the younger generation [24]. The popularity of collaborative research had been attributed to the ease of accessing new information, refinement of communication technologies, reduction in transportation costs, and limitation of funding allowing for the division of costs and resources [25]. The rise in Saudi neurosurgeons’ international collaboration resulted in a reduction in the mean percentage of KSA authorship from 93% to 61% as well a drop in the KSA first authorship rate from 100% to 71%. Naturally, this shift has to be offset against the recognized advantage of international collaboration, which includes the enhancement of the quality of research resulting in higher numbers of scholarly output and higher citation numbers [25].

A change in the distribution of the first authors’ KSA institutions was observed. Over the last decade (2011-2020), compared to the previous two decades (1991-2010), the following institutions saw a drop in their share of first-author publications: KSU dropped from 36.5% to 12.5%, KFSHRC from 16.5% to 8%, IAFU from 19.7% to 3.5%, and AFH from 15.1% to 1%. Over the same period, the following institutions witnessed an increase in their share of publications: KFMC from 0% to 13.5%, KAU from 0.7% to 8% and KAMC from 1.3% to 8%. A change in the distribution of the first authors’ KSA regions was also observed. During 2011-2020, the following regions saw a drop in their share of first-author publications: central from 73.7% to 45%, and the eastern region from 20.4% to 6%. Over the same period, the following regions witnessed an increase in their portion of publications: the western region from 3.3% to 14.5% and the southern region from 0% to 4%.

Over the last decade (2011-2020), there was a significant increase mean study population size from 22.5 to 74. Furthermore, a significant change in the study type was observed with a drop in the case reports rate from 59.2% to 41% and a rise in the reviews rate from 4.6% to 15.5%. Most of the latter were systematic reviews, which are generally associated with a higher level of evidence. The changes are considered favourable and could reflect improvement in study design and quality, which may be associated with a greater impact [4,6,12].

The number of citations an article receives is arguably the most important indicator of its impact and clinical importance [4]. Identification of the predictors of citation rates is useful for researchers and journals in order to boost the impact of their work [4]. In this study, the duration from publication, sample size, and study type were significant predictors of annual citation rates of Saudi publications in the international neurosurgical literature during the whole study period (1991-2020). However, the journal’s IF, number of authors, number of centres, number of countries, and percentage of KSA authorship were positive predictors of annual citation rates for publications during 2011-2020. This reflects that the citation patterns for publications during the last decade (2011-2020) are closer to the findings of others. In recent publications, the duration from publication, LOE, number of centres, number of authors, number of countries, and journal IF were documented as significant predictors of citations for neurosurgical research five years after publication [6].

Limitations

The study had several limitations. It was reliant on the accuracy of data in PubMed and Google Scholar. Some articles may have been missed. The exclusion of publications in neuroscience and medical journals may have influenced the findings. The articles were selected based on having one Saudi neurosurgeon; however, in the calculation of the number of authors, Saudi non-neurosurgeons were included. Focusing on the first author’s centre and region may not reflect the significance of the contribution of the other authors. The influence of the increase in the number of KSA neurosurgeons and the change in country-specific characteristics on productivity during the study period was not examined. There may have been potential errors in the sub-grouping of the study types. The study did not examine the change in trends in the articles' neurosurgical subspecialties. Furthermore, it did not attempt to identify the changes with time in the names of the most productive neurosurgeons or their seniority levels. Article citations were taken at a certain point that was likely to change. The wide study duration may have influenced citations, particularly for older papers. The KSA contribution was not compared to equivalent countries.

## Conclusions

Over the last decade, KSA neurosurgeons’ contribution to international neurosurgical journals has increased considerably. The number of neurosurgeons from new centres and regions that were productive also increased. The publications have become more multi-authored, multi-centred, and multi-national, as well as reporting larger sample sizes and lesser case reports. The changes in publication trends correlate positively with the articles’ annual citation rates. This could be looked upon as a cause for optimism.
